# Nitrogen Depletion Blocks Growth Stimulation Driven by the Expression of Nitric Oxide Synthase in Tobacco

**DOI:** 10.3389/fpls.2020.00312

**Published:** 2020-03-20

**Authors:** Andrés Nejamkin, Noelia Foresi, Martín L. Mayta, Anabella F. Lodeyro, Fiorella Del Castello, Natalia Correa-Aragunde, Néstor Carrillo, Lorenzo Lamattina

**Affiliations:** ^1^Instituto de Investigaciones Biológicas, Facultad de Ciencias Exactas y Naturales, Universidad Nacional de Mar del Plata, Mar del Plata, Argentina; ^2^Instituto de Biología Molecular y Celular de Rosario (IBR-CONICET), Facultad de Ciencias Bioquímicas y Farmacéuticas, Universidad Nacional de Rosario, Rosario, Argentina

**Keywords:** nitric oxide, nitric oxide synthase, *Nicotiana tabacum*, nitrogen, plant growth, seed production

## Abstract

Nitric oxide (NO) is a messenger molecule widespread studied in plant physiology. Latter evidence supports the lack of a NO-producing system involving a NO synthase (NOS) activity in higher plants. However, a NOS gene from the unicellular marine alga *Ostreococcus tauri* (*OtNOS*) was characterized in recent years. *Ot*NOS is a genuine NOS, with similar spectroscopic fingerprints to mammalian NOSs and high NO producing capacity. We are interested in investigating whether OtNOS activity alters nitrogen metabolism and nitrogen availability, thus improving growth promotion conditions in tobacco. Tobacco plants were transformed with *OtNOS* under the constitutive CaMV 35S promoter. Transgenic tobacco plants expressing *OtNOS* accumulated higher NO levels compared to siblings transformed with the empty vector, and displayed accelerated growth in different media containing sufficient nitrogen availability. Under conditions of nitrogen scarcity, the growth promoting effect of the *OtNOS* expression is diluted in terms of total leaf area, protein content and seed production. It is proposed that OtNOS might possess a plant growth promoting effect through facilitating N remobilization and nitrate assimilation with potential to improve crop plants performance.

## Introduction

Crop productivity depends on strong nitrogen (N) fertilization, though plants use only 50% of the supplied nitrogen. For most plant species, NUE is defined by the plant capacity to extract inorganic nitrogen (N) from the soil, assimilate nitrate and ammonium, translocate, remobilize and recycle of organic N forms during the life cycle (Krstić and Sarić, 1983). Metabolic processes based on protein synthesis and N-containing biomolecules are critical for plant vegetative and reproductive growth and yield, and dependent on the adequate N supply (Sinclair and Rufty, 2012). Thus, improving NUE is a big challenge for plant biotechnology (Masclaux-Daubresse et al., 2010).

Both field and laboratory researches have demonstrated that increasing the supply of N fertilizers enhances growth and photosynthesis. The sensitivity for N fertilization is species specific and central for agriculture. Nitrogen scarcity results in a reduced leaf area and leaf energy production due to a reduced light interception for photosynthesis (Huber et al., 1989; Tóth et al., 2002; Dordas and Sioulas, 2008).

Given that nitrate reduction is the rate limiting step for N assimilation, nitrate reductase (NR) is considered a key enzyme in N acquisition. NR reduces nitrate to nitrite, but is also able to generate NO from nitrite (Kaiser et al., 2010). More recently, NR has been shown to play a role in NO homeostasis by supplying electrons from NAD(P)H through its diaphorase/dehydrogenase domain both to a truncated phytoglobins, which scavenges NO by its dioxygenase activity, and to the molybdoenzyme NO-forming nitrite reductase that can also generate NO from nitrite (Chamizo-Ampudia et al., 2017). In addition, a Nitrite:NO reductase was characterized as a membrane–bound enzyme that specifically produces NO from nitrite at pH 6 (Stöhr et al., 2001). It may reduce the apoplastic nitrite produced by NR playing a role in nitrate signaling via NO formation (Stöhr et al., 2001).

NO is a widespread signal molecule that participates in many physiological processes in all life kingdoms. In animals, NO is produced by the enzyme NO synthase (NOS; EC 1.14.13.39). All NOS make use of l-arginine and molecular oxygen as substrates and require the reduced cofactors nicotinamide-adenine-dinucleotide phosphate (NADPH), flavin adenine dinucleotide (FAD), flavin mononucleotide (FMN), and (6*R*-)5,6,7,8-tetrahydrobiopterin (BH_4_). Animal NOS is a bimodal enzyme, comprising an N-terminal oxygenase domain (NOSoxy) that binds protoporphyrin IX (heme) and C-terminal reductase domain (NOSred) that binds NADPH and the cofactors FMN and FAD. The two domains are connected by a calmodulin binding sequence (Griffith, 1995).

Genomic and functional analyses indicate that NOS enzymes are present in many organisms ranging from bacteria to humans (Gorren and Mayer, 2007). In higher plants, there are at least two enzymatic ways leading to NO production, the reductive and oxidative pathways: (1) NR, which reduces nitrate to nitrite, and then nitrite to NO (Yamasaki et al., 1999) and (2) a NOS-like enzymatic activity (Corpas et al., 2006). Nevertheless, no gene or protein with sequence similarity to animal or bacterial NOS has been yet identified in higher plants (Jeandroz et al., 2016). The first NOS of the plant kingdom was described in the photosynthetic unicellular Chlorophyte *Ostreococcus tauri* (Foresi et al., 2010; Weisslocker-Schaetzel et al., 2017). *O. tauri* is a single-celled green alga who shares a common ancestor with higher plants and is considered part of an early diverging class within the green plant lineage. Thus, it is an appropriate model system to study gene evolution and cellular processes in photosynthetic eukaryotes (Derelle et al., 2006).

In a previous work, we expressed *OtNOS* in Arabidopsis under the regulation of a stress-inducible promoter and showed that the transgenic lines displayed improved tolerance against salt and drought stresses (Foresi et al., 2015). The relevance of NO as a mediator of physiological and stress-related processes in plants has recently been reviewed (Begara-Morales et al., 2018). Meanwhile, other reports demonstrated that NO can regulate the first steps of N assimilation (Sanz-Luque et al., 2013) and also, through overexpression of phytoglobins, that NO can be oxidized to NO_3_^–^ and enter into N assimilation pathways in dicots and monocots (Kuruthukulangarakoola et al., 2017; Zhang et al., 2019). In this work, based on the molecular characterization of *Ot*NOS activity and NO involvement in N metabolism, we generated transgenic tobacco plants expressing *OtNOS* under the control of the Cauliflower Mosaic Virus (CaMV) 35S promoter, and analyzed their growth and response to sufficient and deficient N conditions. Transgenic tobacco lines expressing *OtNOS* exhibited higher growth rates than plants transformed with the empty vector (EV) in nitrate sufficient condition. These findings extend our knowledge about the physiology of NO and nitrate in plants.

## Materials and Methods

### Preparation and Characterization of *Nicotiana tabacum* Lines Expressing *OtNOS*

The *O. tauri* DNA sequence encoding NOS was synthesized, sequenced and cloned into the *Bam*HI and *Xba*I sites of pCHF3 (Jarvis et al., 1998) to give pCHF3:*OtNOS*. Plasmid pCHF3 is a binary vector carrying the CaMV 35S promoter and a pea (*Pisum sativum* L.) rubisco small subunit terminator. *Nicotiana tabacum* plants were transformed as described by Gallois and Marinho (1995) using *Agrobacterium tumefaciens* containing the plasmid of interest. Seedlings of *N. tabacum* cultivar Petit Havana (PH) from 6 to 8 weeks grown in Magenta boxes with MS-0, 0.8% (w/v) agar were used. After incubation and transformation process, stems of 1 to 2 cm long were transferred to Magenta boxes with MS-0, 0.8% (w/v) agar containing 0.1 mg ml^–1^ Kanamycin (Kan). Stems that formed roots were subsequently transferred to pots with soil.

Transgenic *35S:OtNOS* lines were selected on the basis of Kan resistance, and confirmed by genomic PCR and RT-PCR analyses. Primers used for RT-PCR are specified in Supplementary Table S1 and amplified a 632-bp fragment. The primers for elongation factor 1α (*ef-1α*), a housekeeping gene used as a reference for mRNA levels between samples, are also indicated in Supplementary Table S1. For quantitative RT-PCR, total RNA was extracted using Trizol (Invitrogen). One μg of total RNA was used for first-strand cDNA synthesis with a M-MLV reverse transcriptase (Promega). PCR reactions were performed using Taq DNA polymerase (Invitrogen) with annealing temperature of 54°C and 35 cycles.

### Plant Growth

Tobacco plants were grown in a culture chamber with long day photoperiod (16:8, light:darkness), temperature of 27°C/23°C (light/dark cycle), 150 μE m^–2^ s^–1^ of light intensity and 60% humidity. For all experiments, transgenic plants of T1 generation were selected by sowing and growing for 9 days in plates containing 0.1 mg.ml^–1^ Kan and 0.8% (w/v) agar supplemented with Hoagland solution (Hoagland and Arnon, 1938) or MS. For experiments under different N conditions, seedlings were transferred to plates containing a modified Hoagland/agar solution without ammonium and with 10 mM NO_3_^–^ (5 mM KNO_3_, 2.5 mM Ca(NO_3_)_2_) or 0.5 mM NO_3_^–^ (KNO_3_). Growth analysis was performed using different N concentrations (10, 3, 1.5, and 0.5 mM) in which 0.5 mM was established as a deficient N condition for tobacco. When nitrate was 0.5 mM, Hoagland solution was supplemented with KCl and CaSO_4_ to maintain Ca^+2^ and K^+^ levels. For experiments with more developed plants, seedlings were transferred to 200-cm^3^ plastic pots with soil:perlite:vermiculite (1:1:1) and irrigated with water or 10 mM KNO_3_. For seed production, seedlings were transferred to 5-lt pots containing perlite:vermiculite (1:1) and irrigated with modified Hoagland solution as previously described.

### Detection of NO and Determination of Nitrate and Protein

Endogenous NO levels were estimated by using the NO-sensitive dye DAF-FM DA (Kojima et al., 1999; Moreau et al., 2008). Roots were observed by fluorescence microscopy and bright-field microscopy using an Eclipse E200 microscopy (Nikon). After Kan selection, seedlings were transferred for 7 days to plates containing agar and modified Hoagland solution as described above. For nitrate and protein determination, 100 mg of seedlings were ground in liquid N_2_ and resuspended in 100 mM sodium phosphate pH 7.4. After centrifugation at 10,000 *g* for 15 min at 4°C, supernatants were used for nitrate determination as described by Cataldo et al. (1975). Samples were incubated with Salicylic acid (50 mg/ml) and reaction was stopped with 2N NaOH. Absorbance was measured at 410 nm. Protein content were determined by the Bradford method (Bradford, 1976).

### Determination of Leaf Area and Root Length

For all treatments, seedlings were photographed, total whole leaf area per plant and total length of all roots per plant were measured using Image J software (Image J^1^).

### Seed Germination

Transgenic tobacco seeds were sown and germinated in plates containing 0.8% (w/v) agar with Hoagland (10 mM NO_3_^–^), prior stratification. Germination was measured after 8 days, when the radicle pierced the seed coat (Bethke et al., 2006).

### Oxygen Consumption

Oxygen consumption of adult leaves was analyzed using a Clark-type oxygen electrode. Detached leaves from tobacco *EV* and transgenic plants from two growth conditions (water and NO_3_^–^), were pre-incubated for 2 h in the dark in the reaction buffer (10 mM K_2_HPO_4_, pH 7.2, 10 mM KCl, 5 mM MgCl_2_, 0.3 M mannitol) before measurements. Then, entire leaves were introduced into the chamber and oxygen consumption was measured for 10 min. Values were normalized to fresh weight and expressed relative to the plants with maximal O_2_ consumption.

### Quantitative PCR Analysis

For quantitative RT-PCR, reactions were performed on a Step-one Real-time PCR machine from Applied Biosystems (California, United States) with Fast Universal SYBR Green Master Rox (Roche) to monitor the synthesis of double-stranded DNA. Software LinReg (Ruijter et al., 2009) was used to analyze data and relative transcript levels for each sample. Data were normalized against the levels of *ef-1*α cDNA. The primer sequences used are listed in Supplementary Table S1. Primers for NR were designed to amplify transcript of both NR isoforms (NIA1, NIA2).

### Segregation Analysis

For estimation of loci number in which T-DNA was integrated, seeds of T1 were sown in Petri plates with MS-0 and Kan and were incubated in a phytotron for 10 days. After that, sensible and resistant plants were counted. It was assumed that if the segregation proportion of Kan resistant: sensible is 3:1, the T-DNA has been inserted in a unique site of the genome (Murgia et al., 2004).

### Statistical Analysis

Results are expressed as means ± standard error. Data were analyzed using the Student’s *t*-test for pairwise comparisons or ANOVA with *post hoc* Dunnett’s method comparisons. We have developed a linear mixed-effects model, using the lme function from the nlme library in R software (version 3.1; R Foundation for Statistical Computing). Fixed effect was *OtNOS* expression, experiments and plates were treated as a random effect.

## Results

### Expression of *OtNOS* Gene in Tobacco

We have previously showed that the expression of *OtNOS* with the control of a stress-inducible short promoter fragment (SPF) of the sunflower (*Helianthus annuus*) *Hahb-4* gene improved stress tolerance of Arabidopsis (Foresi et al., 2015). Based on those results, we tested whether a constitutive expression of *OtNOS* in tobacco plant could also generate a benefit on growth parameters. Thus, *OtNOS* under the control of the constitutive CaMV 35S promoter was cloned into the vector pCHF3 and used to transform tobacco (*Nicotiana tabacum* cv. *Petit Havana*) (Supplementary Figure S1A). Transgenic tobacco T_0_ lines expressing *OtNOS* were screened by genomic PCR (Supplementary Figure S1B) and RT-PCR (Supplementary Figure S1C).

**FIGURE 1:**
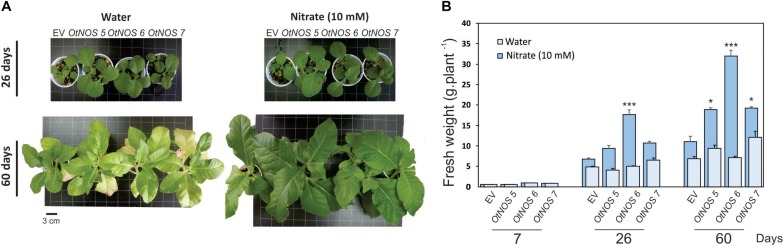
Expression of *OtNOS* promotes growth of transgenic tobacco plants. **(A)** Representative photographs showing phenotypes of tobacco lines transformed with empty vector (*EV*) or *OtNOS* grown in plates containing MS with kanamycin (9 days) and then transferred to pots with substrate (perlite:vermiculite:soil, 1:1:1) and irrigated with water or 10 mM nitrate for the indicated times. **(B)** Fresh weight of *EV* and *OtNOS* transgenic lines was quantified as a measure of vegetative growth in these conditions. Values are means (± SE) of four to five biological replicates. Asterisks indicate statistically significant differences between *OtNOS* lines and *EV* (ANOVA *post hoc* Dunnett’s method was used, *p < 0.1 and ***p < 0.001).

NOS-expressing lines were characterized by measuring fresh weight (FW) of tobacco plants growing in pots containing perlite:vermiculite:soil (1:1:1) irrigated with water or 10 mM nitrate. Transgenic *OtNOS* lines showed higher FW than *EV* plants after 26 and 60 days of irrigating with nitrate, while no significant differences were detected between lines when irrigated with water (Figures 1A,B).

Mitochondria are not only the cell organelles whose tasks are respiration and energy generation, but also they are sites of NO production and target in the electron transport chain (ETC), altering the organelle physiology. The NO production and action on various mitochondrial complexes of the ETC play a major role in NO signaling, energy metabolism and growth of plants (Gupta et al., 2018). To assess whether *OtNOS* expression affects mitochondrial respiration rate, O_2_ consumption was analyzed. We found that leaves of water-watered EV plants had ∼40% more respiration than *OtNOS* lines in the same condition (Supplementary Figure S2). This difference of respiration rates between *OtNOS* and *EV* plants was independent of the age of the plant (40, 60, and 70-day old). In contrast, when plants were supplied with nitrate, less O_2_ consumption was observed with the increasing of the plant age and irrespective of the genotype analyzed (Supplementary Figure S2).

### *OtNOS* Expression Increases Tobacco Seedling Biomass Under N Sufficiency

**FIGURE 2:**
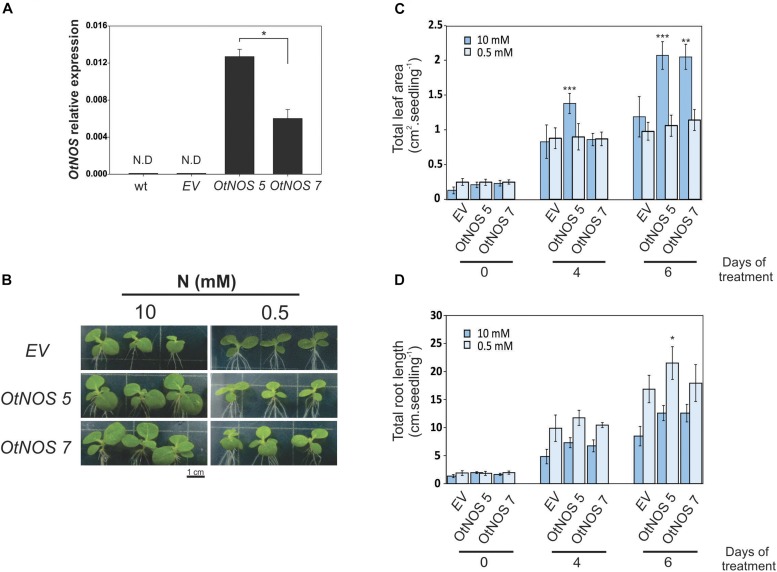
Characterization of transgenic tobacco seedlings expressing *OtNOS* and growing under different N conditions. **(A)** Quantitative RT-PCR analysis of *OtNOS* expression in leaves of transgenic seedlings growing in plates containing Hoagland/agar (10 mM N) for 6 days (previously selected with kanamycin for 9 days). Values are means (± SE) of three independent experiments, each consisting of a biological replicate corresponding to a pool of two to three plants. Values were relativized using *ef1-*α as control. Asterisk indicates statistically significant differences between *OtNOS* lines (Student’s *t*-test, *p < 0.05). N.D, no detection. **(B)** Representative pictures showing the phenotypes of aerial parts of 16-days-old seedlings after 6 days of growing in Hoagland medium containing 10 or 0.5 mM NO_3_^–^. Leaf area **(C)** and total root length **(D)** were measured during treatment using Image J software. Values are means (±SE) of three independent experiments, each consisting of at least three biological replicates. Asterisks indicate statistically significant differences between *OtNOS* lines and seedlings transformed with empty vector (*EV*) in same conditions (ANOVA *post hoc* Dunnett’s method was used, *p < 0.05, **p < 0.01, ***p < 0.001).

Before continuing analyzing transgenic lines, we tested the number of T-DNA insertion (Supplementary Table S2). Since more than one insertion could generate confusing results, the following experiments were done only with the *OtNOS 5* and *OtNOS 7* tobacco transgenic lines. First, we checked *OtNOS* expression by quantitative RT-PCR (qPCR) and observed that *OtNOS 5* levels were two-fold higher than those of *OtNOS 7* (Figure 2A). Since we observed a different growth rate between transgenic and EV plants and considering that OtNOS may affect N metabolism since it uses arginine as substrate, we designed an experiment using Petri dishes containing different N concentrations (10 and 0.5 mM NO_3_^–^). Tobacco seedlings were grown in a modified Hoagland/agar medium in which NO_3_^–^ was used as the sole N source. When seedlings were grown on sufficient NO_3_^–^ concentration (10 mM), the OtNOS expressing seedlings exhibited growth advantages compared with EV (Figure 2B). However, when N was restricted (0.5 mM) this difference was abolished (Figure 2B). Statistical analyses indicate that *OtNOS* expression increased leaf biomass under nitrate-rich conditions and not under N-deficient conditions (Figures 2B,C). To get insights on root growth under contrasting nitrate availability, we measured development of the root system. Results shown in Figure 2D indicate that root length was, as expected, 50% larger under low N since seedling root system tries to explore the growing media seeking for more N (Guan et al., 2014). There are no significant differences in root system growth between EV and *OtNOS* plants except for the transgenic line *OtNOS 5* that displayed a slight increase of root growth under N deficiency at 0.5 mM nitrate (Figure 2D). It has been proved that treatments with exogenous NO donors or complete blockage of endogenous NO modifies root phenotype (Correa-Aragunde et al., 2004). Since no strong effects on root architecture was observed in the transgenic tobacco lines, it is considered that levels of NO present in roots of tobacco *OtNOS* lines are below concentrations required to induce a phenotype.

### Levels of NO in *OtNOS* Transgenic Tobacco Roots

**FIGURE 3:**
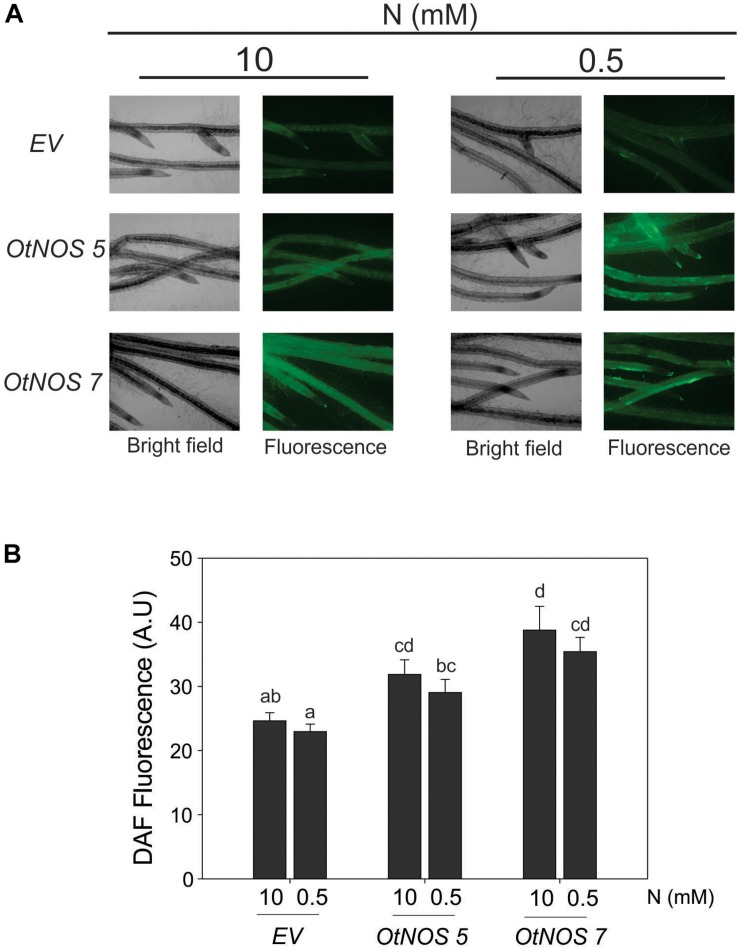
Increased NO content in roots of transgenic tobacco lines expressing *OtNOS*. **(A)** NO content was determined using DAF-FM DA and detected by fluorescence microscopy in roots after 7 days of treatment with 0.5 or 10 mM NO_3_^–^. Images were taken at 40X. **(B)** Quantification of DAF-FM DA fluorescence using Image J software (AU, arbitrary units). Values are means (±SE) of two independent experiments each consisting of three biological replicates. Different letters indicate statistically significant differences (ANOVA, *post hoc* Tukey method was used, *p* < 0.05).

We analyzed if the expression of *OtNOS* leads to an increase in NO production in tobacco plants by using the DAF-FM DA, a cell-permeable fluorescent probe that reacts with the most oxidized forms of NO (NO^+^ and N_2_O_3_). It is the most used probe for NO detection in plants (Guo et al., 2003; Planchet and Kaiser, 2006; Zottini et al., 2007; Lozano-Juste and León, 2010; Foresi et al., 2015). Roots of transgenic tobacco plants incubated with DAF-FM DA were analyzed by epi-florescence microscopy. After treatment, higher green fluorescence was observed in roots of both *OtNOS* transgenic tobacco lines compared to *EV* (Figures 3A,B). *Nicotiana tabacum* corresponds to pattern type 3 of radical hairs distribution, with rows of trichoblasts and trichomes alternating with rows of atrichoblasts (Kim et al., 2006). An increase in the intensity could be seen in the zone of differentiation corresponding to the root zone where trichoblasts and trichomes are generated. However, still the apex zone is observed with fluorescence, which is especially relevant, since NO is required for primary root elongation (Sanz et al., 2014).

Results indicate that constitutive expression of *OtNOS* effectively increased between 25 and 30% the NO production in tobacco root cells. To complement this observation and to get insights of the NO level in transgenic tobacco leaves, qPCR analysis of phytoglobin transcript level was performed considering that its transcript is induced by the increase of NO (Ohwaki et al., 2005; Shimoda et al., 2005; Kuruthukulangarakoola et al., 2017). Phytoglobins are responsible of contributing to the control of high levels of NO in plants (Hebelstrup et al., 2013). In agreement with results obtained using the NO probe in roots, transcript levels of tobacco phytoglobin showed a slight increase in leaves of both transgenic tobacco lines *OtNOS 5* and *OtNOS 7* (Supplementary Figure S3).

### Higher Expression of Nitrate Reductase (*NR*) in Tobacco *OtNOS* Transgenic Line

**FIGURE 4:**
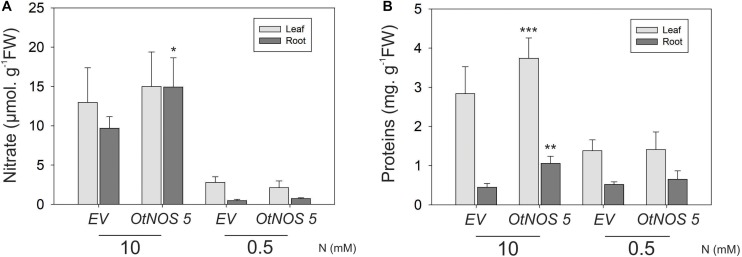
Nitrate and protein content in transgenic tobacco line expressing *OtNOS*. Seedlings were grown in plates with Hoagland/agar medium containing 10 mM NO_3_^–^ and kanamycin for 9 days. After selection, seedlings were transferred to plates with indicated N levels for 7 days for nitrate and protein determinations. Measurements were done using all leaves and root independently. **(A)** Nitrate was measured according to Cataldo et al. (1975). **(B)** Protein content was measured using Bradford’s method (1976). Fresh weight (FW). Values are means (±SE) of at least three independent experiments each consisting of three to four biological replicates corresponding to a pool of two to three plants. Asterisks indicate statistically significant differences between *OtNOS 5* and *EV* (ANOVA, *post hoc* Dunnett’s method was used, *p < 0.05, **p < 0.01, ***p < 0.001).

The growth phenotype of OtNOS transgenic tobacco plants prompted us to further explore N metabolite assimilation in those lines. Nitrate and protein content were measured from leaves and roots of *OtNOS 5* and *EV* tobacco lines. While no differences were found under low N condition, *OtNOS 5* line increases the levels of nitrate and protein compared to *EV* when grown in N sufficient condition (Figure 4). Accordingly, *OtNOS 7* line also increased protein content in leaves and roots during N sufficiency (Supplementary Figure S4).

**FIGURE 5:**
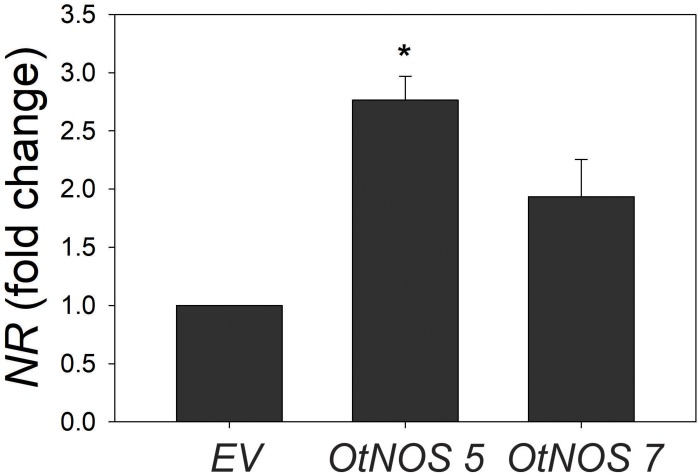
Transcript levels of nitrate reductase (*NR*). Seedlings were grown in plates with Hoagland medium containing 10 mM NO_3_^–^ and kanamycin for 9 days. After selection, seedlings were transferred to plates with the same N condition for 3 days and quantitative RT-PCR analysis of *NR* transcript level was perfomed. *NR* expression was relativized to *EV* plants. Values are means (±SE) of three independent experiments, each consisting of three biological replicates corresponding to a pool of two to three plants. Asterisk indicates statically significant differences compare to EV (Student’s *t*-test, *p < 0.05).

To further investigate nitrate assimilation pathway, levels of NR transcript were studied in a short time of treatment since it is known that genes responding to nitrogen are highly dependent on N availability (Liu et al., 2017). Given that transgenic tobacco lines displayed similar growth phenotype compared to *EV* under low N conditions, we only analyzed NR expression under complete N supply. Results show that NR expression is higher in *OtNOS 5* and *7* compared to *EV* (Figure 5).

### *OtNOS* Expression Might Promote Tobacco Seed Yield When N Is Sufficient

It is well known that seed production is highly dependent on the plant N status (Sinclair and Rufty, 2012). To analyze whether N availability modifies differentially the number of flowers and seed production in transgenic *OtNOS* plants, transgenic and *EV* plants were grown until the end of life cycle. A positive effect of *OtNOS* expression could be observed in the number of flowers and seed yield under complete N condition in the line *OtNOS 5* (Table 1), that correlates with a high level of *OtNOS* expression. This effect was prevented when N supply was deficient (Table 1). Results indicate that N restriction resulted in a severe attenuation of the *OtNOS*-promoted stimulation of growth and production in tobacco.

**TABLE 1:** Transgenic tobacco line *OtNOS5* showed increased seed yield under complete nitrogen supply.

	Nitrogen (mM)	*EV*^a^	*OtNOS 5* ^b^	Ratio ^b/a^	*OtNOS 7* ^c^	Ratio ^c/a^
Flowers. plant^–1^	10 0.5	30 ± 2 16 ± 1	47 ± 4 18 ± 2	1.5** 1.1	33 15 ± 1	1.1 0.9
Total seed weight. plant^–1^ (g)	10 0.5	1.32 ± 0.2 0.64 ± 0.02	2.38 ± 0.5 0.71 ± 0.12	1.8* 1.1	1.0 0.63 ± 0.01	0.8 0.9
Individual seed weight (mg)	10 0.5	0.067 0.058	0.079 0.069	1.2 1.2	0.094 0.068	1.4 1.2
Germination (%)	10 0.5	96 67	98 68	1.02 1.02	78 33	0.81 0.50

Transgenic tobacco plants were grown in 5-lt pots containing perlite:vermiculite (1:1) and irrigated with Hoagland solution containing 10 mM NO_3_^–^ for 40 days. After that, treatment was started by irrigation with Hoagland solution containing 10 mM or 0.5 mM NO_3_^–^ until the end of the plant cycle. Individual area per seed was measured using ImageJ software. All flowers and seeds produced per plants were quantified. Values are means (±SE) of one experiments with two (*OtNOS 7*) and three to four (*EV*, *OtNOS 5*) biological replicates. Statistics were only done between *EV* and *OtNOS 5* transgenic lines. Asterisks indicate statistically significant differences (Student’s *t*-test, *p < 0.1, **p < 0.05).

## Discussion

Optimal plant growth fully relies on the availability of soil nutrients. Nitrogen (N) is a central resource required in large amounts to sustain the synthesis of organic molecules that constitute the plant. Arginine is not only a building block for protein biosynthesis, but also contains the highest nitrogen to carbon (N/C) ratio among amino acids, being a storage molecule of organic N in plant cells. Thus, arginine metabolism possesses many physiological implications in higher plants (Winter et al., 2015). Plant arginases degrade arginine to produce ornithine and urea (Meng et al., 2015). Arginine is also a substrate for NO synthesis in plants through an activity of a yet undescribed protein named NO synthase-like (NOS-like) (Gas et al., 2009). We have previously shown that transgenic expression of *OtNOS* in Arabidopsis resulted in positive effects on germination, aerial growth and responses to water deficit mediated by changes in stomatal index and pore aperture (Foresi et al., 2015).

In this work, we show that transgenic tobacco plants expressing *OtNOS* are able to grow faster than siblings transformed with the *EV*. In addition, the transgenic line *OtNOS 5*, expressing higher level of *OtNOS* than *OtNOS 7* line is able to generate up to 80% more seeds than *OtNOS 7* and *EV* tobacco plants. In another work, transgenic tobacco plants that over expressed a mammalian NOS were generated (Chun et al., 2012). These transgenic plants are smaller compared to the wild type, exhibited enhanced resistance to biotic stress and contained high level of salicylic acid. Mammalian NOS uses the cofactor BH_4_, but there are no biosynthetic pathways described for this cofactor in plants. Unlike OtNOS, that belongs to the plant kingdom and is capable of using tetrahydrofolate (THF) as cofactor, mammalian NOS activity was not detected with THF (Adak et al., 2002). Thereby, more work is necessary to understand how mammalian NOS activity is generating NO in the transformed tobacco plants. Here, it was demonstrated that *OtNOS* expression could bring potential benefits improving N metabolism in tobacco, a plant strongly dependent on N supply (Ruiz et al., 2006). Three experimental conditions were assayed to analyze the effects of *OtNOS* expression on tobacco growing under sufficient and deficient N supply: (*i*) pots with limited soil content supplemented or not with NO_3_^–^, (*ii*) plates containing 0.5 or 10 mM NO_3_^–^ and (*iii*) pots containing perlite:vermiculite (1:1) and irrigated with Hoagland containing 0.5 or 10 mM NO_3_^–^. In all experimental models, *OtNOS* expression conferred tobacco an enhanced growth under sufficient N availability.

Mitochondria are tightly linked to N metabolism and assimilation in plants (Szal and Podgórska, 2012) and is also a source of NO generated from nitrite and cytochrome c oxidoreductase activity in complex III (Alber et al., 2017). Additionally, NO was shown to be able to partially inhibit mitochondrial respiration (Gupta et al., 2018). According to this, under low N condition, *OtNOS* transgenic plants display ∼40% inhibition of mitochondrial respiration respect to *EV* plants. In other reports, it has been shown that NO could inhibit aconitase, induce alternative oxidase (Kumari et al., 2019) and shift the metabolism toward amino acid and protein synthesis (Cvetkovska and Vanlerberghe, 2012; Gupta et al., 2012). We showed that transgenic tobacco lines have no different respiration rates under sufficient N availability. In this sense, it cannot be ascribed OtNOS phenotype to different respiration rates since the increase growth, nitrate and protein content in the *OtNOS* transgenic lines was observed only when plants where supplemented with nitrate.

NR expression was higher in the transgenic line *OtNOS 5* than in *OtNOS 7*, and it correlates with a higher *OtNOS* expression in *OtNOS 5*. NR expression and activity are regulated by many factors, nitrate, light, phytohormones, low temperature, drought, among others (Lillo et al., 2003; Lea et al., 2004; Park et al., 2011). Furthermore, NR is not only a key enzyme for N acquisition and assimilation in plants (Campbell, 2001), but also a key enzyme to modulate plant NO homeostasis (Tejada-Jimenez et al., 2019). The double mutant NR (*nia1 nia2*) has decrease nitrite levels and impaired NO synthesis (Modolo et al., 2006). These evidences indicate that there is a mutual regulation between NR activity and NO, hindering the interpretation of the results.

The NR substrate NO_3_^–^ has also been shown to regulate flowering induction (Marín et al., 2011), crop growth and yield increments (Lawlor, 2002; Krapp et al., 2014). Moreover, the over-expression of tobacco *NR* increases seed protein content and weight in wheat without increasing the N supply (Zhao et al., 2013). We report here that the transgenic tobacco line that express more *OtNOS* transcript levels presented a positive correlation with protein content, NR expression and seed production. However, when N has been restricted, the promoter effect of OtNOS was blocked. Under N deficient conditions, it can be detected similar levels of *OtNOS* expression as in N sufficiency (Supplementary Figure S5) but only a slight decrease in NO content. The lack of correlation between lines for *OtNOS* transcript levels, NO production and the growth phenotype may suggest that multiple factors are involved. Indeed, the effects observed in *OtNOS* lines may be not only due to the increment of NO, but also a result of NOS activity such as arginine depletion and/or increase of citrulline levels. Furthermore, NO homeostasis in plants is regulated through many oxidation processes and enzymatic activities (e.g. phytoglobin activity, chemical reaction with proteins and redox state of the cell).

As stated, NUE is an attractive target to modulate and improve growth and yield in crop plants. Recent findings in cyanobacteria highlight the importance of how N metabolism can be enriched through the activity of new and unexpected enzymes such as a singular NOS with a globin domain found in *Synechoccocus* PCC 7335 (*SyNOS*) (Correa-Aragunde et al., 2018) and arginine dihydrolase found in *Synechocystis* sp. PCC 6803 (Zhang et al., 2018). *SyNOS* and arginine dihydrolase metabolize arginine to generate mainly nitrate and ammonia, respectively, which are able to re-enter in the first steps of N assimilation and supply the elementary blocks required for the synthesis of macromolecules. Increasing the ability of plant cells for recycling macronutrients from storage molecules seems to be a promissory novel strategy to attain the goal of diminishing fertilization practices in crop fields without affecting yield.

## Data Availability Statement

The datasets generated for this study are available on request to the corresponding author.

## Author Contributions

NF, AL, NC-A, NC, and LL are members of the research staff. AN and FD are graduates fellows and MM is a postdoctoral fellow from CONICET, Argentina.

## Conflict of Interest

The authors declare that the research was conducted in the absence of any commercial or financial relationships that could be construed as a potential conflict of interest.

## Footnotes

1 http://imagej.nih.gov/ij/

## Abbreviations

NO: nitric oxide
NUE: nitrogen use efficiency
*Ot*NOS: nitric oxide synthase from *Ostreococcus tauri*.
