# Supplementary material for: Nitrogen Depletion Blocks Growth Stimulation Driven by the Expression of Nitric Oxide Synthase in Tobacco
Source: Front Plant Sci. 2020 Mar 20;11:312. doi: 10.3389/fpls.2020.00312 (PMC7100548; doi:10.3389/fpls.2020.00312)
Supplement: Supplementary file 1 [file Data_Sheet_1.docx]

**[Supporting Information](http://onlinelibrary.wiley.com/enhanced/doi/10.1111/tpj.12471/#footer-support-info)**


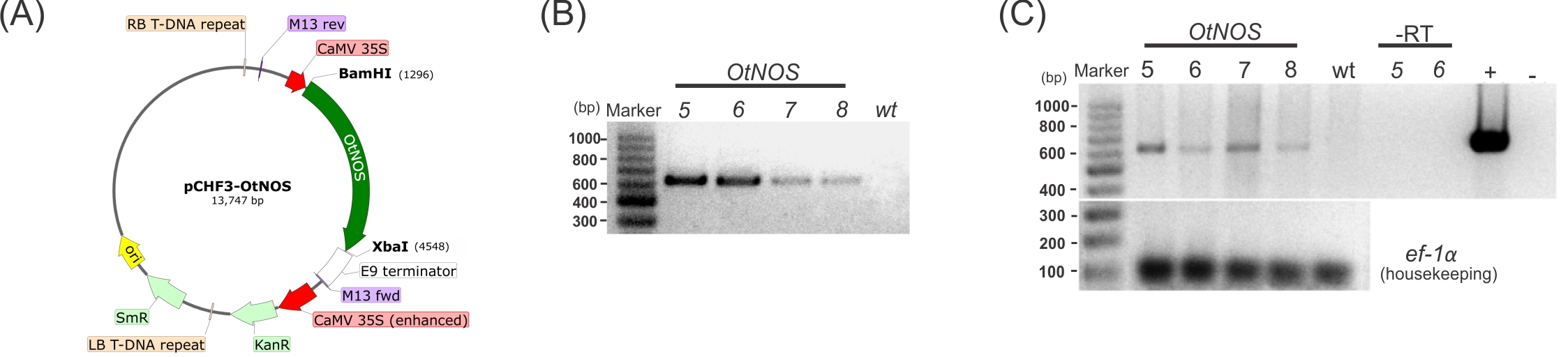


**Figure S1. Transformation of tobacco plants with nitric oxide synthase from *Ostreococcus tauri* (*OtNOS*) and analysis of its expression.** (A) Schematic representation of the constructs used for transformation (done with SnapGene Viewer, https://www.snapgene.com). *OtNOS* was cloned between XbaI and BamHI restriction sites. Terminator from the pea rbcS-E9 gene (E9 Terminator). Sequence for M13 forward and reverse primers (M13 fwd and M13 rev). Cauliflower mosaic virus 35S promoter with or without a duplicated enhancer region (CaMV 35S enhanced and CaMV 35S). Kanamycin resistance (KanR). Right and Left border of T-DNA (RB T-DNA and LB T-DNA). Spectinomycin resistance (SmR). Origin of replication (ori). (B) PCR analysis of genomic DNA of wild type (wt) and independent transgenic lines *OtNOS 5*, *OtNOS 6*, *OtNOS 7* and *OtNOS 8* grown with 10mM NO_3_^-^. (C) RT-PCR analysis of *OtNOS* expression in transgenic lines. Elongation factor 1α (*ef1-α*) cDNA was used as a reference for mRNA transcription. Marker: molecular weight marker. – RT: no RT addition. +,-: positive and negative controls of PCR reaction.


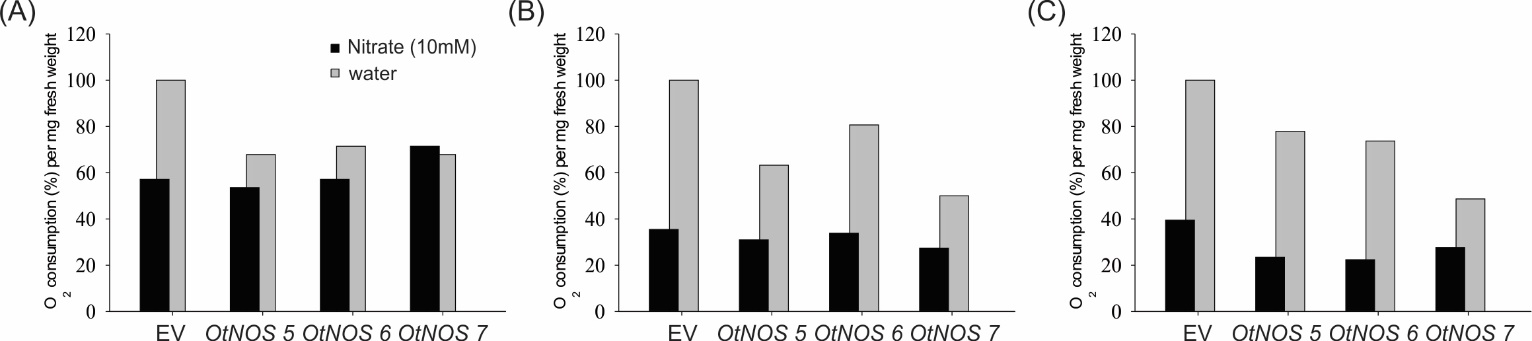


**Figure S2. Oxygen consumption in tobacco transgenic *OtNOS* lines.** (A), (B) and (C) represents oxygen consumption of leaves from 40, 60 and 75 day old plants, respectively, watered with 10 mM NO_3_^-^ or water.


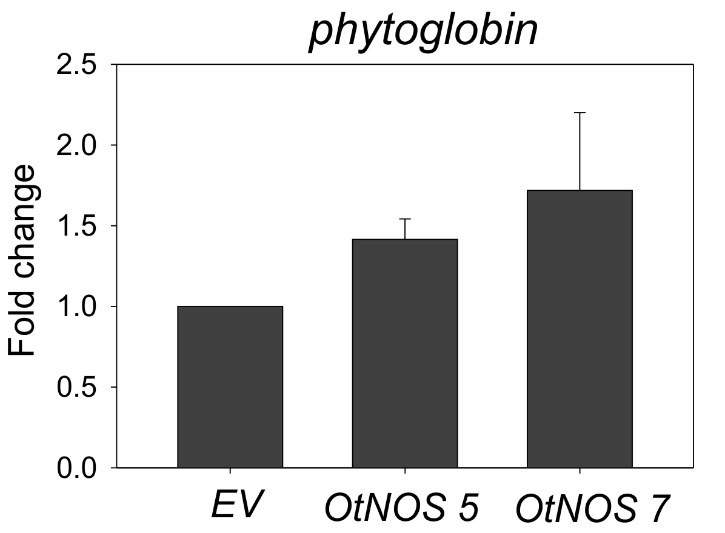


**Figure S3.** Quantitative RT-PCR analysis of *phytoglobin* gene expression. *Phytoglobin* transcript was measured in leaves of transgenic tobacco seedlings expressing *OtNOS* after growing in plates with Hoagland/agar (10 mM NO_3_^-^) for 16 days. Values are means ± SE (n=2) and relativized to *EV*.


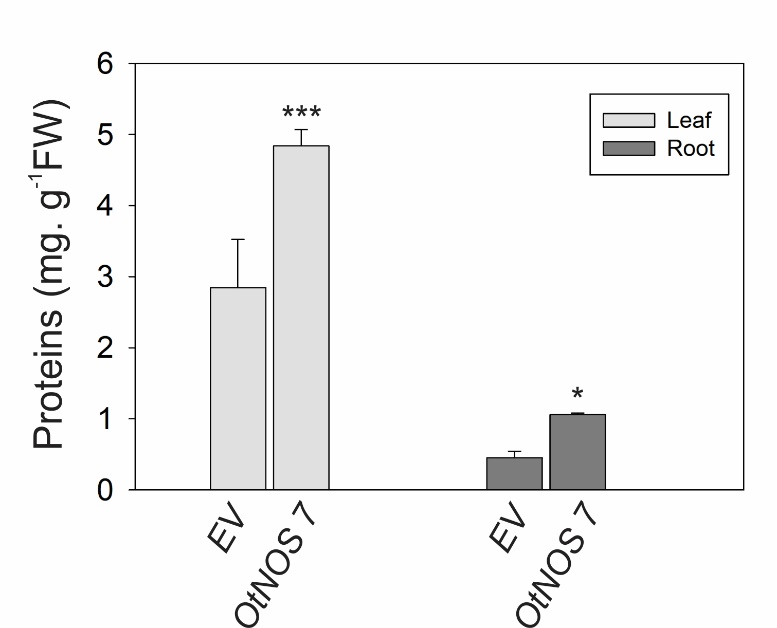


**Figure S4**. Protein content of transgenic tobacco *OtNOS 7* line in sufficient and deficient nitrogen conditions. Transgenic tobacco seedlings were grown for 9 days in plates containing complete Hoagland/agar (10 mM NO_3_^-^) with kanamycin. Then plants were transferred for 7 days to plates containing Hoagland/agar with indicated N level. Asterisks indicate statistically significant differences compared to *EV* in the same condition (lineal mixed model, posthoc Dunnett’s method, *p < 0.1, ***p < 0.01, n =4)


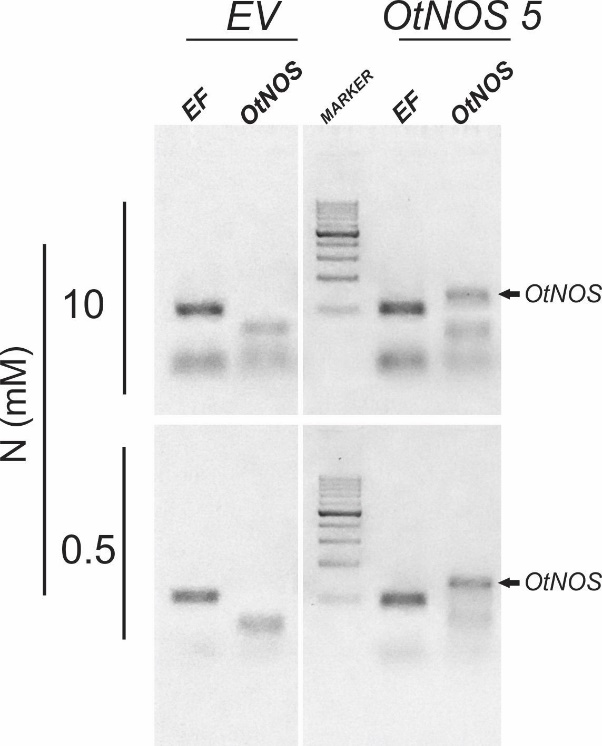


**Figure S5**. OtNOS expression in EV and *OtNOS 5* transgenic line under deficient nitrogen condition. Arrows indicate the size of OtNOS amplicon. Elongation factor (EF) was used as positive control.

| Gene name | Genbank name | | Forward primer 5’- 3’ | Reverse primer 5’ - 3’ |
| --- | --- | --- | --- | --- |
| *OtNOS (1)* | | CAL57731 | CTACACCATGCGGTTGTTTG | CCCTGTGAAGAGTCGAAAGC |
| *OtNOS (2)* | | CAL57731 | TGAGACCGCGCTATTATTCC | CGTAGCGAGAAAATCTGAGC |
| *ef-1α* | | 107826390 | TTCAGGAGCATGCGTCAAACTG | TCTTCTTCTGAGCAGCCTTGGT |
| *NR* | | 107785409 | TGCTGGCACTGATTGCACTG | ATGGACGGAGTTGCCAGGAG |
| *Non-sym Hb* | | 107811444 | TTGCCTTGTTGGAGACAATC | AACTGATCAAAGGCCTCACC |

**Table S1**. Primers list. Primers of *OtNOS (1)* and *OtNOS (2)* were used for RT-PCR and qRT-PCR analysis, respectively.

| Transgenic  lines | Number of plants | | | X^2^ observe  (3:1 segregation) | X^2^obs<X^2^_0.05_  (X^2^_0.05_ = 3.84) | Numbers of  T-DNA insertion |
| --- | --- | --- | --- | --- | --- | --- |
|  | Total | Kan^R^ | Kan^S^ |  |  |  |
| *OtNOS 5* | 191 | 139 | 52 | 0.43 | Yes | 1 |
| *OtNOS 6* | 243 | 217 | 26 | 26.81 | No | >1 |
| *OtNOS 7* | 217 | 267 | 50 | 0.39 | Yes | 1 |
| *OtNOS 8* | 252 | 187 | 65 | 0.08 | Yes | 1 |

**Table S2.** Segregation analysis of kanamycin resistance (Kan^R^) and kanamycin sensitive (Kan^S^) T1 seedlings of transgenic tobacco lines.
